# Determinants of Arbovirus Vertical Transmission in Mosquitoes

**DOI:** 10.1371/journal.ppat.1005548

**Published:** 2016-05-12

**Authors:** Sebastian Lequime, Richard E. Paul, Louis Lambrechts

**Affiliations:** 1 Insect-Virus Interactions Group, Department of Genomes and Genetics, Institut Pasteur, Paris, France; 2 Centre National de la Recherche Scientifique, Unité de Recherche Associée 3012, Paris, France; 3 Université Pierre et Marie Curie, Cellule Pasteur UPMC, Paris, France; 4 Functional Genetics of Infectious Diseases Unit, Department of Genomes and Genetics, Institut Pasteur, Paris, France; University of Alberta, CANADA

## Abstract

Vertical transmission (VT) and horizontal transmission (HT) of pathogens refer to parental and non-parental chains of host-to-host transmission. Combining HT with VT enlarges considerably the range of ecological conditions in which a pathogen can persist, but the factors governing the relative frequency of each transmission mode are poorly understood for pathogens with mixed-mode transmission. Elucidating these factors is particularly important for understanding the epidemiology of arthropod-borne viruses (arboviruses) of public health significance. Arboviruses are primarily maintained by HT between arthropod vectors and vertebrate hosts in nature, but are occasionally transmitted vertically in the vector population from an infected female to her offspring, which is a proposed maintenance mechanism during adverse conditions for HT. Here, we review over a century of published primary literature on natural and experimental VT, which we previously assembled into large databases, to identify biological factors associated with the efficiency of arbovirus VT in mosquito vectors. Using a robust statistical framework, we highlight a suite of environmental, taxonomic, and physiological predictors of arbovirus VT. These novel insights contribute to refine our understanding of strategies employed by arboviruses to persist in the environment and cause substantial public health concern. They also provide hypotheses on the biological processes underlying the relative VT frequency for pathogens with mixed-mode transmission that can be tested empirically.

## Introduction

Host-to-host transmission of pathogens is usually categorized as either vertical or horizontal, irrespective of the physical route of transmission [1]. Vertical transmission (VT), also called hereditary transmission, refers to transmission of a pathogen from parent to offspring. Horizontal transmission (HT) encompasses all other modes of non-parental transmission, including sexual and vector-borne transmission. VT and HT are not mutually exclusive and a combination of both, known as mixed-mode transmission, is common across taxa of hosts and pathogens, including eukaryotes, bacteria, and viruses [2]. Combining both modes of transmission allows pathogens to persist under conditions that would otherwise lead to extinction. Indeed, demographic and epidemiological changes of host populations lead to different opportunities for transmission by one mode or the other. When the density of susceptible hosts is low, for example, VT becomes an essential link in the transmission chain [3]. Elucidating the biological factors governing the relative frequency of HT and VT is essential to understanding the ecology and evolution of pathogens with mixed-mode transmission [2]. This is particularly critical for infectious agents of public health relevance because their epidemiology and control will largely depend on their mode of transmission. Rare VT can be important for persistence of pathogens that would otherwise go extinct during the winter [4]. Such knowledge can be used to prevent VT and consequently reduce the likelihood of future epidemics.

Arthropod-borne viruses (arboviruses) that are pathogenic for humans mainly belong to four distinct genera of RNA viruses: *Alphavirus* (e.g., chikungunya and Eastern equine encephalomyelitis viruses), *Flavivirus* (e.g., Zika, yellow fever, dengue, and West Nile viruses), *Orthobunyavirus* (e.g., California encephalitis virus) and *Phlebovirus* (e.g., Rift Valley fever virus). There are more than 530 described arboviruses, of which about a hundred are pathogenic to humans [5]. Epidemiological cycles of arboviruses often consist of complex transmission networks that involve a variety of vertebrate hosts and hematophagous arthropods, usually referred to as vectors, such as mosquitoes or ticks [6]. Humans are not necessarily at the centre of the transmission network and may only be incidental hosts (e.g., West Nile virus).

Arboviruses are primarily maintained by cross-species transmission between arthropod vectors and vertebrate hosts. Vectors become infected during blood feeding on a viremic vertebrate and, after a period of development within the vector, the virus can infect a new vertebrate host during a subsequent blood meal. This mode of transmission qualifies as HT because it involves unrelated hosts [1]. Although HT largely determines arbovirus epidemiology, some epidemiological features remain unexplained. In particular, the maintenance of arboviruses in endemic areas during adverse conditions for vector activity has long puzzled researchers. Dry seasons in tropical areas, cold seasons in temperate regions, or insecticide spraying campaigns can drastically reduce vector density and thus opportunities for HT [7]. In addition, arbovirus infections in vertebrates usually result in long-lasting protective immunity so that high levels of herd immunity will prevent HT following epidemics. Several hypotheses have been suggested to explain the maintenance of arboviruses during inter-epidemic periods, such as virus re-introduction, circulation in unknown host species (i.e., reservoir), and alternative transmission mechanisms [8]. Phylogeographic studies of dengue virus genomic sequences from isolates collected prior to, during, and post epidemics found that despite a degree of mutation, the virus was the same within each site over time, but different between sites [9,10]. This would suggest that the virus was thus maintained locally despite the lack of conditions permissive to HT.

Arbovirus VT in the arthropod vector population is a proposed maintenance mechanism during adverse conditions for HT. VT is well documented in mosquitoes for several arboviruses across the four aforementioned major viral genera [11]. Arbovirus VT in mosquitoes is generally maternal and occurs through two main mechanisms: transovarial transmission (TOT), whereby the virus infects the germinal tissues of the female mosquitoes, and trans-egg transmission, whereby the virus infects the egg during oviposition [12]. Arboviruses may persist during unfavourable periods through the infection of eggs, larvae, or adults (including diapausing individuals) without the need of HT. The epidemiological significance of arbovirus VT, however, has remained controversial for several arboviruses of public health concern [11]. Controversy primarily stems from the inability of estimated rates of VT to explain long-term arbovirus maintenance, combined with discrepancies between field observations and laboratory studies, as well as inconsistencies among virological techniques [11,13,14]. Here, we used the largest contemporary databases on natural and experimental VT occurrence records to provide a comprehensive list of positive and negative predictors of arbovirus VT in mosquitoes.

Following Clements [15], we defined VT rate as the proportion of vertically infected offspring from a population of infected females. Infection status of mothers is typically unknown in natural settings and rarely assessed in published laboratory experiments. Clements introduced the effective vertical transmission (eVT) rate to describe the proportion of vertically infected offspring, irrespective of the infection status of their mothers [15]. Therefore, eVT is the product of VT rate and infection prevalence in the population of females under consideration. Only when 100% of mothers are infected, such as in most laboratory experiments, does eVT rate equal VT rate. It is worth noting that VT and eVT definitions do not make any assumption about the underlying mechanism (i.e., TOT or trans-egg VT).

## Overview of the Primary Literature

Literature search and database assembly are described elsewhere [11]. Briefly, we previously conducted a systematic review of the literature published prior to 25 September 2013 from various online and physical sources (the full list of publication is provided in S1 Table). We subsequently conducted a “resilience” test to evaluate the impact of publications that may have been missed during the literature search (see below). Literature search was restricted to the three main arboviral families (i.e., *Bunyaviridae*, *Flaviviridae*, and *Togaviridae*). We compiled two databases. Database #1 includes “experimental” VT studies typically conducted in a laboratory setting. Database #2 includes “natural” studies that investigated arbovirus VT in nature by collecting and testing wild, immature male or nulliparous female mosquitoes. Both databases only included publications that specified at least the mosquito and virus species tested, sample size, and detection technique. The full list of variables in databases #1 and #2 can be found in S2 Table.

In natural VT studies, mosquitoes were collected from the field and brought back to the laboratory. Field collections usually consisted of adult males or immature stages (eggs, larvae, or pupae) that were reared in the laboratory to later developmental stages. These were then processed in pools to detect the presence of virus. In experimental VT studies, female mosquitoes from a parental generation were artificially infected (orally, by intra-thoracic [IT] inoculation, or vertically) and subsequently allowed to lay eggs. Eggs were hatched and the offspring sampled at various developmental stages to determine the presence of virus, usually in pools of individuals.

Virological detection techniques belonged to four main categories [11]: animal, cellular, immunological, and molecular assays. In “animal” assays, mosquito extracts were inoculated in vivo into susceptible laboratory animals that were subsequently checked for pathological effects. In “cellular” assays, cytopathological effects were monitored following inoculation of mosquito extracts onto cell cultures in vitro. In “immunological” assays, viral antigens were detected by antibodies with or without previous amplification in cell culture or animal tissues. In “molecular” assays, viral RNA was detected by reverse transcription (RT)-PCR. Although a few studies tested the progeny individually, individual mosquitoes were most often tested in pools. Therefore, the proportion of infected individuals was usually estimated as the minimum infection rate based on the assumption that only one individual was infected in the pool. This assumption is generally reasonable because observed eVT rates are low for arboviruses (see below), but will underestimate VT rate when it is efficient.

In both databases, the *Aedes–Flavivirus* pair was the most represented vector–virus combination (56.7% and 24.6% of all mosquitoes tested in databases #1 and #2, respectively), followed by *Culex–Flavivirus* (34.1% and 43.7% of all mosquitoes tested, respectively). Within the *Aedes–Flavivirus* pair in database #1, dengue viruses (DENV1, DENV2, DENV3, and DENV4) were the most represented viruses (41% of all *Aedes* mosquitoes tested for flavivirus infection and 23% of all mosquitoes tested). Other vector–virus pairs included *Aedes–Orthobunyavirus* (19.5% and 6.1% of all mosquitoes in databases #1 and #2, respectively) and *Aedes–Alphavirus* (4.6% and 1.4% of all mosquitoes tested, respectively).

## Rates of Effective Vertical Transmission (eVT)

**Fig 1:**
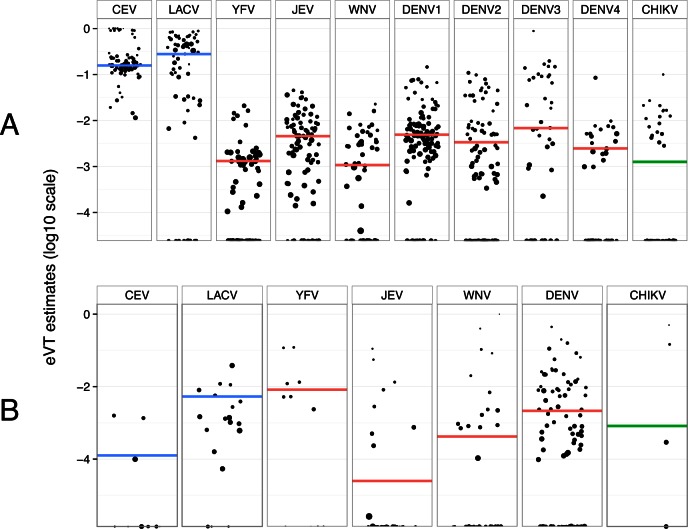
**Distributions of eVT estimates for the most represented arboviruses in (A) experimental and (B) natural studies.** Each data point represents the log_10_-transformed eVT rate obtained from a single database entry. Dot size is proportional to log_10_-transformed sample size. A horizontal coloured line shows the weighted mean eVT for each virus (blue: orthobunyaviruses; red: flaviviruses; green: alphaviruses). CEV: California encephalitis virus; LACV: LaCrosse virus; YFV: yellow fever virus; JEV: Japanese encephalitis virus; WNV: West Nile virus; DENV1-4: dengue viruses; CHIKV: chikungunya virus.

In experimental studies, estimates of eVT rates were significantly higher for the orthobunyaviruses California encephalitis virus (CEV) and LaCrosse virus (LACV) than for flaviviruses or alphaviruses (Fig 1A). CEV and LACV had weighted mean eVT rates of 16% and 28%, respectively, whereas weighted mean eVT rates were 1‰ for yellow fever virus (YFV), 4‰ for Japanese encephalitis virus (JEV), 1‰ for West Nile virus (WNV), 2%–6‰ for DENV1-4, and 1‰ for chikungunya virus (CHIKV). eVT rate estimates were up to several orders of magnitude smaller in natural studies than in experimental studies (Fig 1B). Weighted mean eVT rates in natural studies were 0.1‰ for CEV, 5‰ for LACV, 8‰ for YFV, 0.02‰ for JEV, 0.4‰ for WNV, 2‰ for DENV1-4, and 0.8‰ for CHIKV.

**Fig 2:**
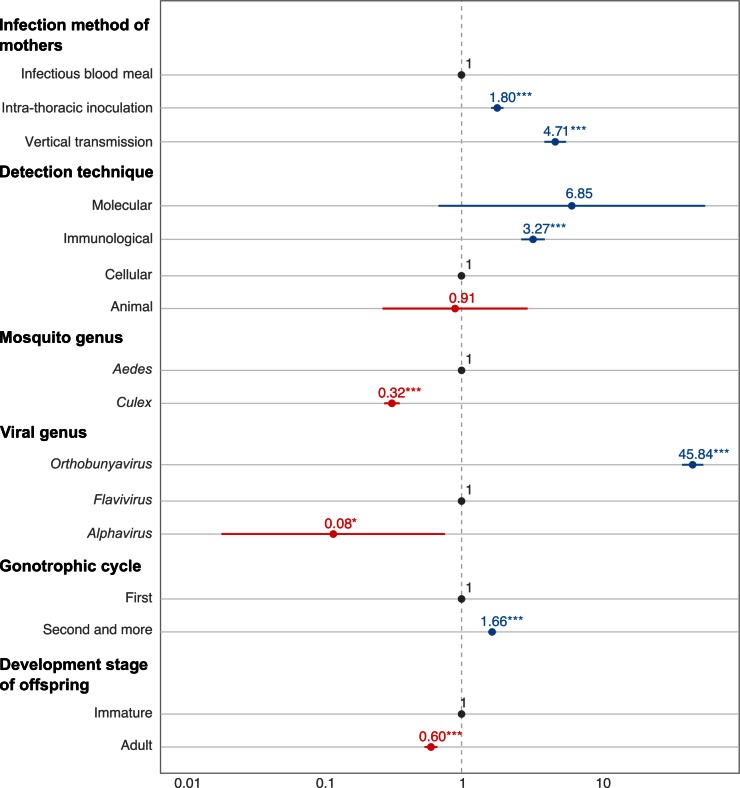
Significant predictors of arbovirus eVT rates in experimental studies. Odds ratios (ORs) and their 95% confidence intervals are shown on a log_10_ scale for statistically significant factors. ORs were calculated from a marginal logistic regression based on a generalized linear mixed model that included the random effect of the study and fixed effects of other covariates. Reference level is shown in grey, positive effects are shown in blue, and negative effects are shown in red. Stars represent statistical significance levels: * *p* < 0.05; ** *p* < 0.01; *** *p* < 0.001.

Mosquito and virus taxonomic groups were strong predictors of the observed level of VT in experimental studies (Fig 2). Mosquitoes in the *Culex* genus were associated with significantly lower eVT rates than mosquitoes in the *Aedes* genus (odds ratio [OR] = 0.32, *p* < 0.001, 95% CI [0.28–0.36]). Using flaviviruses as the reference, viruses of the *Orthobunyavirus* genus had significantly higher eVT rates (OR = 45.71, *p* < 0.001, 95% CI [38.34–54.49]), whereas members of the *Alphavirus* genus had significantly lower eVT rates (OR = 0.08, *p* < 0.01, 95% CI [0.14–0.45]). The method used to infect mothers significantly influenced eVT rate estimates (Fig 2). Both IT inoculated and vertically infected mothers resulted in higher eVT rates (OR = 1.80, *p* < 0.001, 95% CI [1.63–2.00] and OR = 4.71, *p* < 0.001, 95% CI [3.94–5.64], respectively) compared to orally infected mothers. eVT rate estimates also depended on virological assays (Fig 2). Using cellular assays as the methodological reference, only immunological assays were associated with higher eVT rates (OR = 3.27, *p* < 0.001, 95% CI [2.70–3.98]). Offspring produced during the second or later gonotrophic cycles displayed higher eVT rates than offspring produced during the first gonotrophic cycle (OR = 1.66, *p* < 0.001, 95% CI [1.55–1.77]). Lower eVT rates were found when the virus was detected at the adult stage as opposed to immature stages of the progeny (OR = 0.60, *p* < 0.001, 95% CI [0.54–0.67]).

The *Aedes* genus is divided into several subgenera [16], among which *Stegomyia* (including, for example, *Aedes aegypti* and *Aedes albopictus*) and *Ochlerotatus* (including, for example, *Aedes dorsalis*) are the most represented in the VT literature. Because *Stegomyia* mosquitoes are typically associated with flaviviruses, whereas *Ochlerotatus* mosquitoes are typically associated with orthobunyaviruses, the strong effect of the viral genus observed (Fig 2) might be confounded by the mosquito subgenera within the *Aedes* genus. A separate analysis focused on the two *Aedes* subgenera and the two viral genera, *Orthobunyavirus* and *Flavivirus*, showed that their interaction did not significantly influence eVT rates (*p* = 0.09), confirming that orthobunyaviruses had higher eVT rates independently of the mosquito subgenera.

Because the systematic review process to assemble our databases may have missed some publications, we conducted a “resilience” test to evaluate the impact of missing primary data on the outcome of the analyses. We examined the effect of including four publications that we subsequently identified as missing from the original databases [17–20]. Running the same statistical analyses with the four additional publications did not significantly alter the ORs, their CIs, or associated *p*-values (S3 Table). Likewise, it did not change the conclusions from the separate analysis that focused on the two *Aedes* subgenera and the two viral genera, *Orthobunyavirus* and *Flavivirus* (*p* = 0.25). According to this resilience test, a few additional publications do not lead to meaningful differences in our conclusions, owing to the large size of the databases.

## Vertical Transmission of Dengue Viruses

**Fig 3:**
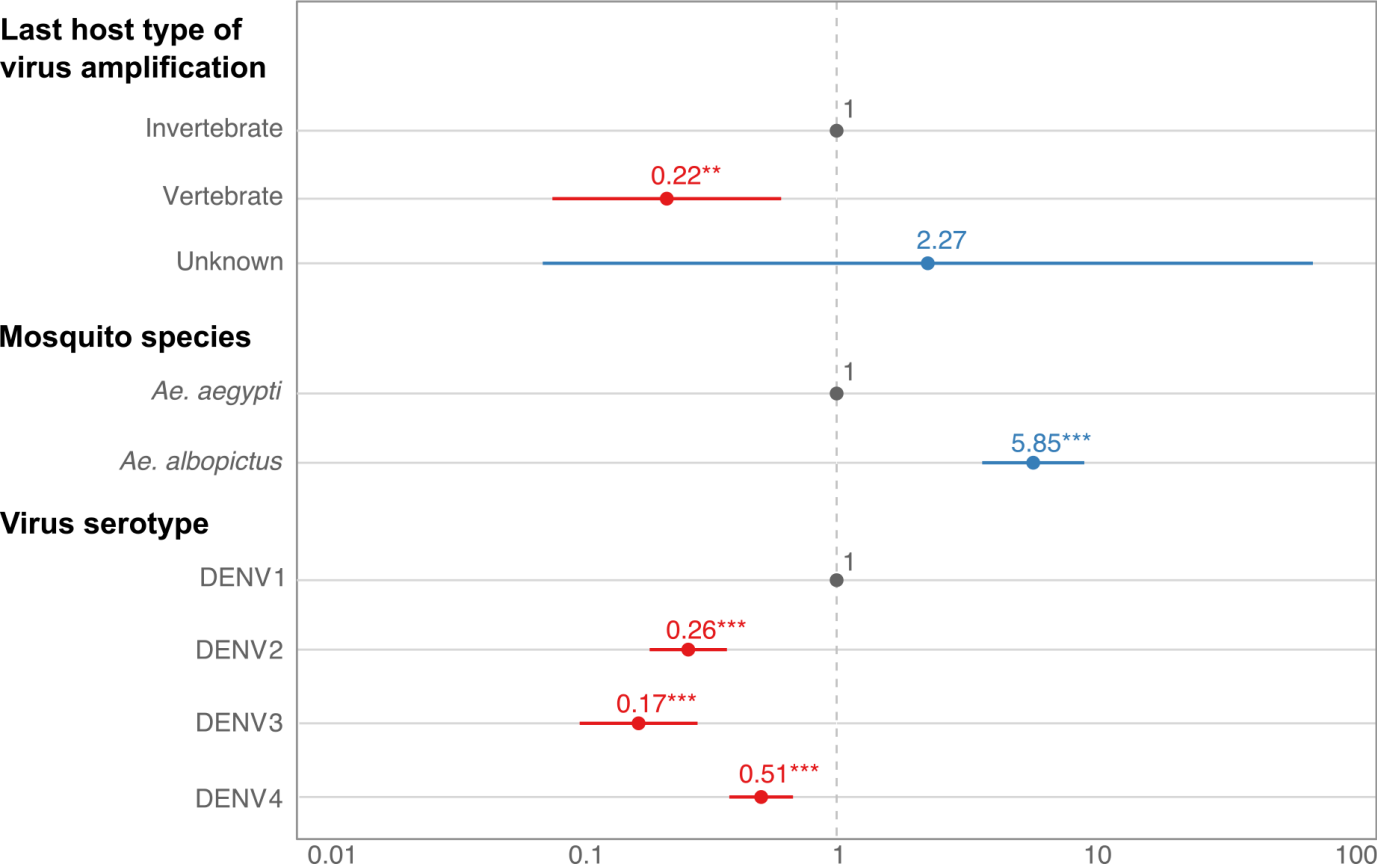
Significant predictors of DENV eVT rates in experimental studies. Odds ratios (ORs) and their 95% confidence intervals are shown on a log_10_ scale for statistically significant factors. ORs were calculated from a marginal logistic regression based on a generalized linear mixed model that included the random effect of the study and fixed effects of other covariates. Reference level is shown in grey, positive effects are shown in blue, and negative effects are shown in red. Stars represent statistical significance levels: * *p* < 0.05; ** *p* < 0.01; *** *p* < 0.001.

DENV1-4 were the most represented viruses in database #1, which allowed more in-depth analyses of the two main DENV vectors worldwide, *Ae*. *aegypti* and *Ae*. *albopictus*. DENV isolates amplified in vertebrate cells (in vivo or in vitro) resulted in lower eVT rates than virus isolates amplified in invertebrate cells (OR = 0.21, *p* < 0.001, 95% CI [0.11–0.38]) (Fig 3). Significantly higher eVT rates were observed in *Ae*. *albopictus* compared to *Ae*. *aegypti* mosquitoes (OR = 5.65, *p* < 0.001, 95% CI [3.57–8.93]). DENV2, DENV3, and DENV4 had significantly lower eVT rates than DENV1 (OR = 0.28, *p* < 0.001, 95% CI [0.20–0.40]; OR = 0.18, *p* < 0.001, 95% CI [0.11–0.31]; and OR = 0.55, *p* < 0.001, 95% CI [0.42–0.73], respectively). DENV4 had significantly higher eVT rates than DENV2 and DENV3 (OR = 0.51, *p* < 0.01, 95% CI [0.34–0.79]; OR = 0.33, *p* < 0.001, 95% CI [0.18–0.58]).

## Climate and Vertical Transmission

Climatic information (main climate, precipitation, and temperature) about each study site was obtained from published data [21] using the Köppen-Geiger climate classification. Study sites from database #2 were geo-localized with Google Earth [22] and layered with climatic data for the 1976–2000 period (available online http://koeppen-geiger.vu-wien.ac.at/shifts.htm), which corresponded to the majority of publications.

**Fig 4:**
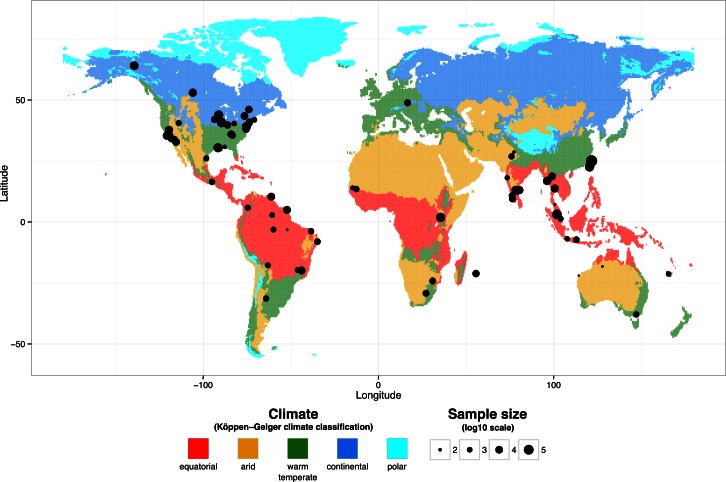
Geographical distribution of main climate types and study sites for natural studies of arbovirus VT in mosquitoes. Climatic data were obtained from Rubel and Kottek (2010) for the 1975–2000 period. Dots represent geographic location of study sites. Dot size is proportional to the log_10_-transformed number of mosquitoes tested in the corresponding study.

**Fig 5:**
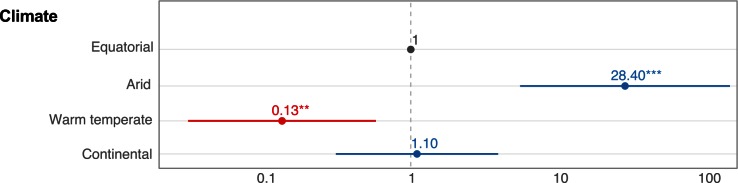
Significant predictors of arbovirus eVT rates in *Aedes* mosquitoes in natural studies. Odds ratios (ORs) and their 95% confidence intervals are shown on a log_10_ scale for statistically significant factors. ORs were calculated from a marginal logistic regression based on a generalized linear mixed model that included the random effect of the study and fixed effects of other covariates. Reference level is shown in grey, positive effects are shown in blue, and negative effects are shown in red. Stars represent statistical significance levels: * *p* < 0.05; ** *p* < 0.01; *** *p* < 0.001.

Most of the natural studies were conducted in Asia, North America, and South America, whereas only a few studies were conducted in Europe and Africa. Fig 4 shows the geographical distribution of VT studies in natural populations overlaid with the main climatic regions defined according to the Köppen-Geiger classification. Overall, statistical power to detect differences was low in database #2 due to exceedingly small eVT rates. Moreover, data structure was highly clustered because of multicollinearity between climate classification and several other variables. For instance, viruses in the *Orthobunyavirus* genus were exclusively associated with warm temperate and continental climate types. Therefore, statistical analyses were performed on selected subsets of data to minimize confounding effects. A subset of the database focusing on arbovirus VT in *Culex* mosquitoes did not reveal any significant predictor. Another subset focusing on arbovirus VT in *Aedes* mosquitoes showed higher eVT rates in mosquito–virus pairs found under arid climate (OR = 28.40, *p* < 0.001, 95% CI [5.52–146.25]) and lower eVT rates under warm temperate climate (OR = 0.13, *p* < 0.01, 95% CI [0.03–0.58]) with equatorial climate as the reference (Fig 5). Note that statistically significant associations between climate type and eVT rates represent overall trends and many counterexamples exist. A final subset focusing on *Flavivirus* VT found a significantly lower eVT rate in adults compared to when immature stages were tested (OR = 0.60, *p* < 0.05, 95% CI [0.40–0.91]).

## Discussion

We reviewed the abundant published literature on arbovirus VT in mosquitoes to identify determinants of VT rates in a case study of pathogens with mixed-mode transmission. We used the largest contemporary databases of natural and experimental VT occurrence records to identify several biological factors associated with arbovirus VT in mosquitoes. Our previous study, in which we assembled the databases, focused on historical and technological aspects of arbovirus VT research [11]. In particular, we found that the probability of VT detection changed with the evolution of virological assays, with enhanced eVT detection associated with increased assay sensitivity and larger sample sizes [11]. In the present review, we used the databases to examine a broader array of biological factors associated with arbovirus VT in mosquitoes. Our analyses provide new insights into the biology of arboviruses and, more generally, into the epidemiology of pathogens with mixed-mode transmission.

Our analysis found striking differences in eVT rates across virus and mosquito taxa. Overall, *Aedes* mosquitoes displayed higher eVT rates than *Culex* mosquitoes. *Aedes* eggs are generally more resistant to desiccation than *Culex* eggs [23], which may confer a selective advantage to vertically transmitted viruses. In addition, *Aedes* mosquitoes had higher eVT rates in nature under arid climatic conditions compared to equatorial or warm temperate climatic conditions. This supports the hypothesis that VT could be a maintenance mechanism during the adverse season. In agreement with earlier observations [7,24,25], orthobunyaviruses such as LACV and CEV were vertically transmitted better than flaviviruses and alphaviruses. Orthobunyaviruses are assumed to achieve higher VT rates because they are vertically transmitted by TOT, a more efficient VT mechanism than trans-egg VT of flaviviruses [12]. It is noteworthy that the *Flavivirus* genus includes several insect-specific flaviviruses that are primarily maintained by VT in nature [26]. Unlike dual-host flaviviruses [12], insect-specific flaviviruses display high rates of VT that are assumed to result from TOT [27], suggesting that different VT mechanisms may have evolved in the same viral genus. TOT relies on viral infection of developing oocytes, which results in close to 100% infection in the subsequent generations [24]. Such “stabilized” infections have been described for several members of the *Orthobunyavirus* genus such as CEV in *Ae*. *dorsalis* [28] and San Angelo virus in *Ae*. *albopictus* [29]. Note that even when TOT rates are close to 100%, prevalence in the vector population is expected to remain low unless the virus confers an evolutionary advantage to the infected subpopulation. TOT may not be required for trans-generational persistence of arboviral infection in the vector, as patterns consistent with stabilized infections were also reported for the flaviviruses DENV1 in *Ae*. *albopictus* [30] and DENV3 in *Ae*. *aegypti* [31]. A fourfold increase in DENV3 eVT rate indicated that selection could be involved [31]. Interestingly, we found that prior DENV amplification in invertebrate host cells enhanced subsequent eVT, supporting the idea that stabilized infections require adaptation. Because systemic dissemination of the virus acquired by HT during blood feeding is a prerequisite for VT, stabilized infections are also more likely to be established during later gonotrophic cycles. VT efficiency could be underestimated in studies that focus on the first gonotrophic cycle and therefore do not account for stabilized infections [24]. When a stabilized infection is established, however, VT is expected to occur as soon as the first gonotrophic cycle.

Our analyses support the idea that arbovirus VT efficiency largely depends on the interplay between gonotrophic cycle and viral infection dynamics. Offspring produced during the second or later gonotrophic cycles displayed higher eVT rates than offspring produced during the first gonotrophic cycle following the infectious blood meal. This can be attributed to the lack of virus dissemination to the ovaries before the first batch of eggs is produced and laid, and/or increased permeability to virus of the ovaries during oogenesis [32]. In addition, the experimental method to infect females was a significant predictor of eVT rates in their progeny. IT inoculations, which bypass the natural infection route through the gut, resulted in higher eVT rates than oral infections. This is presumably because IT-inoculated mothers more quickly develop a systemic viral infection and reach higher viral titers than orally infected females [33]. Vertically infected mothers also had higher eVT rates, which could be a consequence of a stabilized infection of the germ line [24]. Surprisingly, eVT rates measured in immature developmental stages were higher than in the corresponding adults, although the underlying mechanism is unclear. *Aedes aegypti* vertically infected with YFV [34], Kunjin virus, and JEV [35] have delayed development and vertically infected larvae may also suffer lower survival, hence leading to lower infection prevalence in adults.

Some of our findings have important implications for arbovirus–mosquito pairs of public health significance. For instance, DENV had significantly higher eVT rates in *Ae*. *albopictus* than in *Ae*. *aegypti* mosquitoes. Although this has been previously suggested in the literature [36,37], reports have been conflicting [38]. This finding raises important concerns for potential maintenance of arboviruses in areas where *Ae*. *albopictus* has recently expanded geographically, including temperate zones where VT would enable the virus to overwinter [39]. There is currently a critical lack of field and laboratory studies that have examined DENV VT in European *Ae*. *albopictus* populations. Our analysis also revealed that eVT rates in *Aedes* mosquitoes varied considerably among DENV serotypes, as was recently hypothesized [14]. Specifically, DENV1 was significantly better transmitted vertically than the other DENV serotypes. Although there is no obvious explanation for this phenomenon, accounting for such a serotype-specific feature could improve dengue epidemiological models. Such genetic variation could fuel adaptive evolution of VT under a scenario where vectors move into more seasonally hostile areas.

Evolution and maintenance of VT as part of a mixed-mode transmission strategy is expected to be under strongest selective pressure when the horizontal route is limiting. For arboviruses, this will occur when there is periodicity in host (arthropod or vertebrate) abundance, whether due to seasonal climate forcing, herd immunity, or boom-bust cycles of population densities. Imposed seasonality in vector population densities will inevitably select for VT irrespective of the number of competent vector species and thus VT would be expected to be under increasing selection along a latitudinal gradient. Depending on the extent of population genetic structure of vector species and the distribution of the virus, an intra-specific latitudinal cline of VT frequency might occur, and thus VT would not necessarily be a fixed characteristic of a virus–vector couple. Likewise, the lack of sufficient susceptible hosts may be alleviated when viruses have multiple vertebrate host species. Thus, VT might be expected less likely to occur in generalists than specialists. Finally, the selective advantage of a mixed mode of transmission will be limited by the duration of infection and life expectancy of the vertebrates and vectors. Maintaining a reservoir of infection in one or the other is the key, and thus evolution towards persistent infection in the vertebrate host would offer a viable alternative to the risky VT route where egg mortality due to abiotic factors will likely be high. The complete gamut of possibilities may not be available for all pathogens because of the specific physiologies of their hosts; thus, identifying the ecological factors selecting for VT may be best approached within a phylogenetic framework. VT is expected to favour co-divergence of hosts and pathogens [40]. Co-phylogenetic relationships have been suggested between *Aedes* mosquitoes of the *Ochlerotatus* subgenus and orthobunyaviruses in North America [41]. However, this was not supported by our analyses, which failed to find a statistically significant effect of the *Orthobunyavirus–Ochlerotatus* pairings on experimental eVT rates.

In conclusion, our review uncovered a variety of environmental (e.g., climate), taxonomic (e.g., viral genus), and physiological (e.g., gonotrophic cycle) predictors of arbovirus eVT rate. The influence of experimental factors such as the infection route or the mosquito developmental stage tested calls for caution in interpreting results generated from different experimental designs. Our results emphasize the fact that arbovirus VT efficiency is a dynamic process that may vary within and between mosquito generations. Further studies are needed to determine whether permanent germ line infection in so-called stabilized infections could contribute to long-term arbovirus maintenance in a vector subpopulation [24,28]. Ultimately, this knowledge could help to prevent VT and reduce the risk of arbovirus transmission. More generally, our study provides empirically testable hypotheses to investigate the biological processes underlying the relative frequency of each transmission mode for pathogens with mixed-mode transmission.

## Methods

Databases #1 and #2 are provided as supporting information in S4 and S5 Tables, respectively. All quantitative analyses were performed in the statistical environment R, version 3.2.0 (http://www.r-project.org/), using the following packages: plyr [42], ggplot2 [43], stringr [44], gridExtra [45], car [46], lme4 [47], multcomp [48], sjPlot [49], and arm [50].

We computed the mean eVT rate for the most represented arboviruses in each database based on eVT rate estimates for each entry in the database weighted by its sample size. Prevalence is usually close to 100% in experimental studies and, therefore, eVT rate is a reasonable proxy for VT rate. Variation in eVT rate estimates was analysed using generalized linear mixed models (GLMMs). Publication was included as random-effect variable in the GLMMs to account for the collective effect of several potentially confounding variables (experimenter, laboratory conditions, etc.). All other variables were considered as fixed-effect variables. For each entry in the database, the numbers of vertically infected and uninfected individuals were calculated from the eVT rate estimate and the sample size. These numbers were analysed as a contingency table by fitting a model with a binomial error distribution, a logit link function, and a bobyqa optimizer with a maximum of 100,000 function evaluations.

Multicollinearity between model variables was evaluated using the variance inflation factor (VIF) and the condition number (CN). A VIF < 4 and a CN < 30 were interpreted as a low level of multicollinearity [51,52]. Validity of the models was checked using quantile-quantile (Q-Q) plots of random effects and binned residuals average against expected values plots. The Ω^2^_0_ measure of explained variation for mixed-effects models [53] was calculated as a simple goodness-of-fit metric. Following model validation, statistically significant effects (*p* < 0.05) were determined by analysis of deviance and type II Wald χ^2^ statistics. Statistically insignificant variables were removed from the model in a stepwise fashion, repeating model validation at each step. The minimum adequate model (MAM) was obtained when all variables had a statistically significant effect (*p* < 0.05) according to the type II Wald χ^2^ test. Odds ratios (ORs) were calculated based on estimated regression coefficients of the MAM. OR confidence intervals and *p*-values were computed using estimated regression coefficients and their standard errors.

## Supporting Information

S1 Table Full list of publications included in databases #1 and #2. Grey background indicates studies that tested mosquitoes individually for VT.
(DOCX)

S2 Table Full list of factors recorded in databases #1 and #2. (DOCX)

S3 Table Resilience test. Comparison of odds ratios, confidence intervals, and *p*-values before and after addition of four missing publications.
(DOCX)

S4 Table Database #1. (CSV)

S5 Table Database #2. (CSV)
