# Supplementary material for: Determinants of Arbovirus Vertical Transmission in Mosquitoes
Source: PLoS Pathog. 2016 May 12;12(5):e1005548. doi: 10.1371/journal.ppat.1005548 (PMC4865232; doi:10.1371/journal.ppat.1005548)
Supplement: S1 Table — Grey background indicates studies that tested mosquitoes individually for VT. (DOCX) [file ppat.1005548.s001.docx]

| **First author and publication year** | **Title** | **Journal or book** | **Study type** |
| --- | --- | --- | --- |
| Ahmad 1997 | Detection of dengue virus from field Aedes aegypti and Aedes albopictus adults and larvae | Southeast Asian J Trop Med Pub Health | Natural |
| Aitken 1979 | Transovarial transmission of yellow fever virus by mosquitoes (Aedes aegypti) | Am J Trop Med Hyg | Experimental |
| Akbar 2008 | PCR detection of dengue transovarial transmissibility in Aedes aegypti in Bandung, Indonesia. | Proc ASEAN Congr Trop Med Parasitol | Natural |
| Anderson 2006a | West Nile virus from female and male mosquitoes (Diptera: Culicidae) in subterranean, ground, and canopy habitats in Connecticut | J Med Entomol | Natural |
| Anderson 2006b | Importance of vertical and horizontal transmission of west nile virus by culex pipiens in the Northeastern United States | J Infect Dis | Natural |
| Anderson 2008 | Extrinsic incubation periods for horinzontal and vertical transmission of West Nile Virus by Culex pipiens pipiens (Diptera: Culicidae) | J Med Entomol | Experimental |
| Anderson 2012 | Horizontal and vertical transmission of West Nile virus genotype NY99 by Culex salinarius and genotypes NY99 and WN02 by Culex tarsalis | Am J Trop Med Hyg | Experimental |
| Andreadis 2010 | Studies on hibernating populations of Culex pipiens from a West Nile virus endemic focus in New York City: parity rates and isolation of West Nile virus | J Am Mosq Control Asso | Natural |
| Andrews 1977 | Isolation of trivittatus virus from larvae and adults reared from field-collected larvae of Aedes trivittatus (Diptera: Culicidae) | J Med Entomol | Natural |
| Angel 2008b | Distribution and seasonality of vertically transmitted dengue viruses in Aedes mosquitoes in arid and semi-arid areas of Rajasthan, India | J Vector Borne Dis | Natural |
| Arunachalam 2002 | Vertical transmission of Japanese encephalitis virus in Mansonia species, in an epidemic-prone area of southern India | Ann Trop Med Parasitol | Natural |
| Arunachalam 2008 | Natural vertical transmission of dengue viruses by Aedes aegypti in Chennai, Tamil Nadu, India | Indian J Med Res | Natural |
| Bailey 1978 | Isolation of St. Louis encephalitis virus from overwintering Culex pipiens mosquitoes | Science | Natural |
| Balfour 1975 | Isolates of California encephalitis (LaCrosse) virus from field collected eggs and larvae of Aedes triseriatus: identification of the overwintering site of California encephalitis | J Infect Dis | Natural |
| Baqar 1993 | Vertical transmission of West Nile virus by Culex and Aedes species mosquitoes | Am J Trop Med Hyg | Experimental |
| Bardos 1975 | Isolation of Tahyna virus from field collected Culiseta annulata (Schrk.) larvae | Acta Virol | Natural |
| Bardos 1978 | Virological examination of mosquito larvae from southern Moravia | Folia Parasitol | Natural |
| Beaty 1975 | Emergence of La Crosse virus from endemic foci | Am J Trop Med Hyg | Natural |
| Beaty 1980 | Transovarial transmission of yellow fever virus in Stegomyia mosquitoes | Am J Trop Med Hyg | Experimental |
| Bellini 2012 | Impact of Chikungunya virus on Aedes albopictus females and possibility of vertical transmission using the actors of the 2007 outbreak in Italy | PLoS One | Experimental |
| Belloncik 1982 | Activity of California encephalitis group viruses in Entrelacs (province of Quebec, Canada) | Can J Microbiol | Natural |
| Berry 1974 | Isolation of LaCrosse virus (California encephalitis group) from field collected Aedes triseriatus (Say) larvae in Ohio (Diptera: Culicidae) | Mosq News | Natural |
| Berry 1977 | Evidence for transovarial transmission of Jamestown canyon virus, in Ohio | Mosq News | Natural |
| Bina 2008 | Natural vertical transmission of dengue virus in peak summer collections of Aedes aegypti (Diptera: Culicidae) from Urban Areas of Jaipur (Rajasthan) and Delhi | J Commun Dis | Natural |
| Bosio 1992 | Variation in the efficiency of vertical transmission of dengue-1 virus by strains of Aedes albopictus (Diptera: Culicidae). | J Med Entomol | Experimental |
| Broom 1995 | Two possible mechanisms for survival and initiation of Murray Valley encephalitis virus activity in the Kimberley region of western Australia | Am J Trop Med Hyg | Natural |
| Bugbee 2004 | The discovery of West Nile virus in overwintering Culex pipiens (Diptera: Culicidae) mosquitoes in Lehigh County, Pennsylvania | J Am Mosq Control Asso | Natural |
| Campbell 1991 | Isolation of Jamestown Canyon virus from boreal Aedes mosquitoes from the Sierra Nevada of California | Am J Trop Med Hyg | Natural |
| Cecilio 2009 | Natural vertical transmission by Stegomyia albopicta as dengue vector in Brazil | Braz J Biol | Natural |
| Chamberlain 1957 | The North American arthropod-borne encephalitis viruses in Culex tarsalis Coquillett. | Am J Hyg | Experimental |
| Chamberlain 1959 | St. Louis encephalitis virus in mosquitoes | Am J Hyg | Experimental |
| Chamberlain 1964 | Studies on transovarial transmission of St. Louis encephalitis virus by Culex quiquefasciatus Say. | Am J Hyg | Experimental |
| Chen 1990 | A study on transovarial transmission of dengue type I virus in Aedes aegypti | Chinese J Microbiol Immunol | Experimental |
| Chen 2010 | Screening of dengue virus in field-caught Aedes aegypti and Aedes albopictus (Diptera: Culicidae) by one step SYBR Green-based reverse transcriptase-polymerase chain reaction assay during 2004-2007 in Southern Taiwan | Vector Borne Zoonotic Dis | Natural |
| Christensen 1978 | Laboratory studies of transovarial transmission of trivittatus virus by Aedes trivitattus. | Am J Trop Med Hyg | Experimental |
| Clark 1982 | Lacrosse virus activity in Illinois detected by ovitraps | Mosq News | Natural |
| Clark 1983 | Persistence of La Crosse virus (California encephalitis serogroup) in north-central Illinois | Am J Trop Med Hyg | Natural |
| Clark 1985 | Absence of eastern equine encephalitis (EEE) virus in immature Coquillettidia perturbans associated with equine cases of EEE | J Am Mosq Control Asso | Natural |
| Corner 1980 | Cache Valley virus: experimental infection in Culiseta inornata. | Can J Microbiol | Experimental |
| Cornet 1979 | Une poussée épizootique de Fièvre jaune selvatique au Sénégal oriental. Isolement du virus de lots de Moustiques adultes m‚les et femelles | Med Mal Infect | Natural |
| Cornet 1984 | Dengue 2 au Sénégal oriental : une poussée épizootique en milieu selvatique ; isolements du virus à partir de moustiques et d'un singe et considérations épidémiologiques | Cah ORSTOM, sÈr Ent mÈd Parasitol | Natural |
| Crane 1977 | Transovarial transmission of California encephalitis virus in the mosquito Aedes dorsalis at Blue Lake, Utah | Mosq News | Natural |
| Danielova 1979 | Laboratory demonstration of transovarial transmission of Tahyna virus in Aedes vexans and the role of this mechanism in overwintering of this arbovirus | Folia Parasitol | Experimental |
| Davies 1954a | Observations on the biology of West Nile virus, with special reference to its behavior in the mosquito Aedes aegypti | Ann Trop Med Parasitol | Experimental |
| Davis 1930 | The location of yellow fever virus in infected mosquitoes and the possibility of hereditary transmission | Am J Epidemiol | Experimental |
| de Castro 2004 | Dengue virus detection using reverse transcription-polymerase chain reaction in salive and progeny of experimentally infected Aedes albopictus from Brazil | Mem Inst Oswaldo Cruz | Experimental |
| de Souza 1991 | Vertical transmission of dengue 1 virus by Haemagogus equinus mosquitoes | J Am Mosq Control Asso | Experimental |
| Delatte 2008 | in Aedes albopictus, vecteur des virus du chikungunya et de la dengue à la Réunion : biologie et contrôle | Parasite | Natural |
| Dhanda 1989 | Japanese encephalitis virus infection in mosquitoes reared from field-collected immatures and in wild-caught males | Am J Trop Med Hyg | Natural |
| Dhileepan 1996 | Evidence of Vertical Transmission of Ross River and Sindbis Viruses (Togaviridae: Alpha virus) by Mosquitoes (Diptera: Culicidae) in Southeastern Australia | J Med Entomol | Natural |
| Diallo 2000 | Vertical transmission of the yellow fever virus by Aedes aegypti (Diptera, Culicidae): dynamics of infection in F1 adult progeny of orally infected females | Am J Trop Med Hyg | Experimental |
| Dohm 2002 | Experimental vertical transmission of West Nile virus by Culex pipiens (Diptera: Culicidae) | J Med Entomol | Experimental |
| Dutary 1981 | Transovarial transmission of yellow fever virus by a sylvatic vector, Haemagogus equinus. | Trsn Roy Soc Trop Med Hyg | Experimental |
| Eastwood 2011 | West Nile virus vector competency of Culex quiquefasciatus mosquitoes in the Galapagos Islands | Am J Trop Med Hyg | Experimental |
| Farajollahi 2005 | Detection of West Nile viral RNA from an overwintering pool of Culex pipiens pipiens (Diptera: Culicidae) in New Jersey, 2003 | J Med Entomol | Natural |
| Fauran 1990 | Etude sur la transmission verticale des virus de la dengue dans le Pacifique Sud | Bull Soc Path Ex | Natural |
| Flores 2010 | Vertical transmission of St. Louis encephalitis virus in Culex quiquefasciatus (Diptera:Culicidae) in Cordoba, Argentina | Vector Borne Zoonotic Dis | Both |
| Fontenille 1997 | First evidence of natural vertical transmission of yellow fever virus in Aedes aegypti, its epidemic vector | Trsn Roy Soc Trop Med Hyg | Natural |
| Fontenille 1998 | La transmission verticale du virus amaril et ses consÈquences | International Seminar on Yellow Fever in Africa | Natural |
| Fouque 1996 | Aedes aegypti en Guyane française : quelques aspects de l'histoire, de l'écologie générale et de la transmission verticale des virus de la dengue | Bull Soc Path Ex | Natural |
| Fouque 2004 | Epidemiological and entomological surveillance of the co-circulation of DEN-1, DEN-2 and DEN-4 viruses in French Guiana | Trop Med Int Health | Natural |
| Francy 1981 | Transovarial transmission of St. Louis encephalitis virus by Culex pipiens complex mosquitoes | Am J Trop Med Hyg | Experimental |
| Freier 1984 | Oral and transovarial transmission of La Crosse virus by Aedes atropalpus | Am J Trop Med Hyg | Experimental |
| Freier 1987 | Vertical transmission of dengue virus by mosquitoes of the Aedes scutellaris complex mosquitoes. | Am J Trop Med Hyg | Experimental |
| Freier 1988 | Vertical transmission of dengue viruses by Aedes mediovitattus | Am J Trop Med Hyg | Experimental |
| Fulhorst 1994 | Natural vertical transmission of western equine encephalomyelitis virus in mosquitoes | Science | Natural |
| Gargan 1988 | Panveld oviposition sites of floodwater Aedes mosquitoes and attempts to detect transovarial transmission of Rift Valley fever virus in South Africa | Med Vet Entomol | Natural |
| Gillett 1950 | Experiments to test the possibility of transovarial transmission of yellow fever virus in the mosquito Aedes (Stegomyia) africanus Theobald | Ann Trop Med Parasitol | Experimental |
| Goddard 2003 | Vertical transmission of West Nile virus by three California Culex (Diptera: Culicidae) species | J Med Entomol | Experimental |
| Gokhale 2001 | Vertical transmission of dengue-2 through Aedes albopictus mosquitoes | J Commun Dis | Experimental |
| Gottfried 2002 | Temporal abundance, parity, surivaval rates and arbovirus isolation of field-collected container-inhabiting mosquitoes in eastern Tennessee | J Am Mosq Control Asso | Natural |
| Graham 1999 | Selection of refractory and persmissive strains of Aedes triseriatus (Diptera: Culicidae) for transovarial transmission of La Crosse virus | J Med Entomol | Experimental |
| Guedes 2010 | Patient-based dengue virus surveillance in Aedes aegypti from Recife, Brazil | J Vector Borne Dis | Natural |
| Günther 2007 | Evidence of vertical transmission of dengue virus in two endemic localities in the state of Oaxaca, Mexico. | Intervirol | Natural |
| Hardy 1980 | Effect of rearing temperature on transovarial transmission of St. Louis encephalitis virus in mosquitoes. | Am J Trop Med Hyg | Experimental |
| Hardy 1984 | Experimental transovarial transmission of St. Louis encephalitis virus by Culex and Aedes mosquitoes | Am J Trop Med Hyg | Both |
| Hartanti 2010 | Dengue virus transovarial transmission by Aedes aegypti | Univ Med | Natural |
| Hayes 1962 | Detection of eastern encephalitis virus and antibody in wild and domestic birds in Massachusett | Am J Hyg | Natural |
| Hughes 2006 | Comparative potential of Aedes triseriatus, Aedes albopictus, and Aedes aegypti (Diptera: Culicidae) to transovarially transmit La Crosse virus. | J Med Entomol | Experimental |
| Hull 1984 | Natural transovarial transmission of dengue 4 virus in Aedes aegypti in Trinidad | Am J Trop Med Hyg | Natural |
| Hutamai 2007 | A survey of dengue viral infection in Aedes aegypti and Aedes albopictus from re-epidemic areas in the North of Thailand using nucleic acid sequence based amplification assay. | Southeast Asian J Trop Med Pub Health | Natural |
| Ibanez-Bernal 1997 | First record in America of Aedes albopictus naturally infected with dengue virus during the 1995 outbreak at Reynosa, Mexico | Med Vet Entomol | Natural |
| Ilkal 1991 | Entomological investigations during outbreaks of dengue fever in certain villages in Maharashra state | Indian J Med Res | Natural |
| Joshi 1996 | Transovarial transmission of dengue 3 virus by Aedes aegypti | Trsn Roy Soc Trop Med Hyg | Both |
| Joshi 2002 | Persistence of Dengue-3 virus through transovarial transmission passage in successive generations of Aedes aegypti mosquitoes | Am J Trop Med Hyg | Experimental |
| Jousset 1981 | Geographic Aedes aegypti strains and dengue-2 virus: susceptibility, ability to transmit to vertebrate and transovarial transmission | Ann Virol (Inst Pasteur) | Experimental |
| Jupp 1981 | Laboratory vector studies on six mosquito and on tick species with chikungunya virus. | Trsn Roy Soc Trop Med Hyg | Experimental |
| Jupp 1990 | Ae. Furcifer and other mosquitoes as vectors of Chikungunya virus at Mica, northeastern Transvaal, South Africa | J Am Mosq Control Asso | Natural |
| Kappus 1982 | La Crosse virus infection and disease in Western North Carolina | Am J Trop Med Hyg | Natural |
| Kay 1980 | Transovarial transmission of Murray Valley encephalitis virus by Aedes aegypti (L). | Aust J Exp Biol Med Sci | Experimental |
| Kay 1982 | Three modes of transmission of Ross River virus vy Aedes vigilax (Skuse). | Aust J Exp Biol Med Sci | Experimental |
| Khin 1983 | Transovarial transmission of dengue 2 virus by Aedes aegypti in nature | Am J Trop Med Hyg | Natural |
| Kow 2001 | Detection of dengue viruses in field caught male Aedes aegypti and Aedes albopictus (Diptera: Culicidae) in Singapore by type-specific PCR | J Med Entomol | Natural |
| Kumari 2013 | First indigenous transmission of Japanese Encephalitis in urban areas of National Capital Territory of Delhi, India | Trop Med Int Health | Natural |
| Labuda 1983 | Experimental model of transovarial transmission of Tahyna virus in Aedes aegypti | Acta Virol | Experimental |
| Le Goff 2011 | Natural vertical transmission of dengue viruses by Aedes aegypti in Bolivia | Parasite | Natural |
| LeDuc 1975 | Ecology of California encephalitis viruses on the Del Mar Va Peninsula. II. Demonstration of transovarial transmission | Am J Trop Med Hyg | Natural |
| Lee 1997 | Does transovarial transmission of dengue virus occur in Malaysian Aedes aegypti and Aedes albopictus? | Southeast Asian J Trop Med Pub Health | Experimental |
| Lee 2005 | Transovarial transmission of Dengue virus in Aedes aegypti and Aedes albopictus in relation to dengue outbreak in an Urban area in Malaysia | Dengue Bull | Natural |
| Lindsay 1993 | Ross River virus isolations from mosquitoes in Arid Regions of Western Australia: Implication of vertical transmission as a means of persistence of the virus | Am J Trop Med Hyg | Natural |
| Linthicum 1985 | Rift Valley fever virus (family Bunyaviridae, genus Phlebovirus). Isolations from Diptera collected during an inter-epizootic period in Kenya | J Hyg | Natural |
| Lisitza 1977 | Prevalence rates of LaCrosse virus (California encephalitis group) in larvae from overwintered eggs of Aedes triseriatus | Mosq News | Natural |
| McAbee 2008 | Identification of Culex pipiens complex mosquitoes in a Hybrid zone of West Nile virus transmission in Fresno county, California | Am J Trop Med Hyg | Natural |
| McLean 1977 | Natural foci of California encephalitis virus activity in the Yukon territory | Can J Public Health | Natural |
| McLintock 1976 | Isolation of snowshoe hare virus from Aedes implicatus larvae in Saskatchewan | Mosq News | Natural |
| Micieli 2013 | Vector competence of Argentine mosquitoes (Diptera: Culicidae) for West Nile virus (Flaviviridae: Flavivirus). | J Med Entomol | Experimental |
| Miller 1977 | Vertical transmission of La Crosse virus (California encephalitis group): transovarial and filial infection rates in Aedes triseriatus (Diptera: Culicidae) | J Med Entomol | Experimental |
| Miller 1979 | Aedes triseriatus and La Crosse virus: lack of infection in eggs of the first ovarian cycle following oral infection of females | Am J Trop Med Hyg | Experimental |
| Miller 1982 | Variation of La Crosse virus filial infection rates in geographic strains of Aedes triseriatus (Diptera: Culicidae) | J Med Entomol | Experimental |
| Miller 2000 | First field evidence for natural vertical transmission of West Nile virus in Culex univittatus complex mosquitoes from Rift Valley province, Kenya | Am J Trop Med Hyg | Natural |
| Mishra 2001 | Transovarial transmission of West Nile virus in Culex vishnui mosquito. | Indian J Med Res | Experimental |
| Mitamura 1950 | Seasonal occurrence of mosquito in Okayama 1946 and infectivity of the mosquito with Japanese B encephalitis virus; trans ovary infection of the virus in mosquito | Jap Med J | Experimental |
| Mitchell 1990a | Vector competence of Aedes albopictus for a newly recognized Bunyavirus from mosquitoes collected in Potosi, Missouri. | J Am Mosq Control Asso | Experimental |
| Mitchell 1990b | Vertical transmission of dengue viruses by strains of Aedes albopictus recently introduced into Brazil. | J Am Mosq Control Asso | Experimental |
| Mondet 2002 | Isolation of yellow fever virus from nulliparous Haemagogus janthinomys in Eastern Amazonia | Vector Borne Zoonotic Dis | Natural |
| Morris 1978 | An Evaluation of the Hypothesis of Transovarial transmission of Eastern Equine encephalomyelitis virus by Culiseta melanura | Am J Trop Med Hyg | Natural |
| Mourya 1987a | Experimental transmission of Chikungunya virus by Aedes vittatus mosquitoes. | Indian J Med Res | Experimental |
| Mourya 1987b | Absence of transovarial transmission of chikungunya virus in Aedes aegypti & Ae. albopictus mosquitoes. | Indian J Med Res | Experimental |
| Mourya 2001 | Horizontal and vertical transmission of dengue virus type 2 in highly and lowly susceptible strains of Aedes aegypti mosquitoes | Acta Virol | Experimental |
| Mulyatno 2012 | Vertical transmission of dengue virus in Aedes aegypti collected in Surabaya, Indonesia, during 2008-2011 | Jpn J Infect Dis | Natural |
| Muul 1975 | Ecological studies of Culiseta melanura (Diptera:Culicidae) in relation to eastern and western equine encephalomyelitis viruses on the eastern shore of Maryland | J Med Entomol | Natural |
| Nasci 2001 | West Nile virus in overwintering Culex mosquitoes, New York City 2000 | Emerg Infect Dis | Natural |
| Nayar 1986 | Experimental vertical transmission of Saint Louis encephalitis virus by Florida mosquitoes. | Am J Trop Med Hyg | Experimental |
| Nelms 2013a | Experimental and natural vertical transmission of West Nile virus by California Culex (Diptera: Culicidae) mosquitoes | J Med Entomol | Experimental |
| Nelms 2013b | Phenotypic variation among Culex pipiens complex (Diptera: Culicidae) populations from the Sacramento Valley, California: horizontal and vertical transmission of West Nile virus, diapause potential, autogeny, and host selection. | Am J Trop Med Hyg | Experimental |
| Nir 1963 | Failure to obtain experimental transovarian transmission of West Nile virus by Aedes aegypti | Ann Trop Med Parasitol | Experimental |
| Pantuwatana 1974 | Isolation of La Crosse virus from field collected Aedes triseriatus larvae | Am J Trop Med Hyg | Natural |
| Paulson 1989 | Replication and dissemination of La Crosse virus in the competent vector Aedes triseriatus and the incompetent vector Aedes hendersoni and evidence for transovarial transmission by Aedes hendersoni (Diptera: Culicidae) | J Med Entomol | Experimental |
| Pelz 1990 | Vertical transmission of St Louis encephalitis virus to autogenously developed eggs of Aedes atroplapus mosquitoes | J Am Mosq Control Asso | Experimental |
| Pessoa Martins 2012 | Occurrence of Natural Vertical transmission of Dengue-2 and Dengue-3 viruses in Aedes aegypti and Aedes albopictus in Fortaleza, Ceara, Brazil | PLoS One | Natural |
| Philip 1929 | Possibility of hereditary transmission of yellow fever virus by Aedes aegypti (Linn.) | J Exp Med | Experimental |
| Philipps 2006 | Field-caught Culex erythrothorax larvae found naturally infected with West Nile virus in Grand county, utah. | J Am Mosq Control Asso | Natural |
| Pinger 1983 | Isolation of La Crosse and other arboviruses from Indiana mosquitoes | Mosq News | Natural |
| Pinheiro 2005 | Detection of dengue virus serotype 3 by reverse transcription polymerase chain reaction in Aedes aegypti (Diptera: Culicidae) captured in Manaus, Amazonas | Mem Inst Oswaldo Cruz | Natural |
| Ramalingam 1986 | Does transovarial transmission of dengue virus occur in Malaysia | Trop Biomed | Natural |
| Reese 2010 | Identification of super-infected Aedes triseriatus mosquitoes collected as eggs from the field and partial characterization of the infecting La Crosse viruses | Virol J | Natural |
| Reeves 1946 | Laboratory transmission of Japanese B encephalitis virus by seven species (three genera) of North America mosquitoes | J Exp Med | Experimental |
| Reisen 2006 | Overwintering of West Nile virus in Southern California | J Med Entomol | Both |
| Rohani 2007 | Detection of transovarial dengue virus from field-caught Aedes aegypti and Ae albopictus larvae using C6/36 cell culture and reverse transcriptase polymerase chain reaction (RT-PCR) techniques | Dengue Bull | Natural |
| Rohani 2008 | Persistency of transovarial dengue virus in Aedes aegypti (Linn.) | Southeast Asian J Trop Med Pub Health | Experimental |
| Romero-Vivas 1998 | Determination of dengue virus serotypes in individual Aedes aegypti mosquitoes in Colombia | Med Vet Entomol | Natural |
| Rosen 1978 | Transovarial transmission of Japanese encephalitis virus by mosquitoes | Science | Experimental |
| Rosen 1980 | Transovarial transmission of Japanese encephalitis virus by Culex tritaeniorhynchus mosquitoes. | Am J Trop Med Hyg | Experimental |
| Rosen 1983 | Transovarial transmission of dengue virus by mosquitoes: Aedes albopictus and Aedes aegypti | Am J Trop Med Hyg | Experimental |
| Rosen 1987 | Mechanism of vertical transmission of the dengue virus in mosquitoes | C R Acad Sciences | Experimental |
| Rosen 1988 | Further observation on the mechanism of vertical transmission of flaviviruses by Aedes mosquitoes | Am J Trop Med Hyg | Experimental |
| Rosen 1989a | A longitudinal study of the prevalence of Japanese encephalitis virus in adult and larval Culex tritaeniorhynchus mosquitoes in northern Taiwan | Am J Trop Med Hyg | Natural |
| Rosen 1989b | Experimental vertical transmision of Japanese encephalitis virus by Culex tritaeniorhynchus and other mosquitoes | Am J Trop Med Hyg | Experimental |
| Scherer 1986 | Vector incompetency: its implication in the disappearance of epizootic Venezuelan equine encephalomyelitis virus from Middle America | J Med Entomol | Experimental |
| Schopen 1991 | Vertical and veneral transmission of California group viruses by Aedes triseriatus and Culiseta inornata mosquitoes | Acta Virol | Experimental |
| Scott 1990 | Susceptibility of Aedes albopictus to infection with eastern equine encephalomyelitis virus | J Am Mosq Control Asso | Experimental |
| Serufo 1993 | Isolation of dengue virus type 1 from larvae from Aedes albopictus in Campos Altos city, state of Minas Gerais, Brazil | Mem Inst Oswaldo Cruz | Natural |
| Shroyer 1986a | Transovarial maintenance of San Angelo virus in sequential generations of Aedes albopictus | Am J Trop Med Hyg | Experimental |
| Shroyer 1990 | Vertical maintenance of dengue-1 virus in sequential generations of Aedes albopictus | J Am Mosq Control Asso | Experimental |
| Soman 1985 | Transovarial transmission of Japanese encephalitis virus in Culex bitaeniorhynchus mosquitoes | Indian J Med Res | Experimental |
| Sprance 1981 | Experimental evidence against the transovarial transmission of eastern equine encephalitis virus in Culiseta melanura | Mosq News | Both |
| Stamm 1962 | Arbovirus studies in south Alabama, 1957-1958 | Am J Hyg | Natural |
| Stockes 1928 | Experimental transmission of yellow fever to laboratory animals | Am J Trop Med Hyg | Experimental |
| Sudeep 2013 | Preliminary findings on Bagaza virus (Flavivirus: Flaviviridae) growth kinetics, transmission potential & transovarial transmission in three species of mosquitoes. | Indian J Med Res | Experimental |
| Takashima 1989 | Horizontal and Vertical transmission of Japanese Encephalitis Virus by Aedes japonicus (Diptera: Culicidae) | J Med Entomol | Experimental |
| Tesh 1975 | Laboratory studies of transovarial transmission of La Crosse and other arboviruses by Aedes albopictus and Culex fatigans | Am J Trop Med Hyg | Experimental |
| Tesh 1980a | Experimental studies on the transovarial transmission of Kunjin and San Angelo viruses in mosquitoes | Am J Trop Med Hyg | Experimental |
| Tesh 1980b | The mechanism of arbovirus transovarial transmission in mosquitoes: San Angelo virus in Aedes albopictus | Am J Trop Med Hyg | Experimental |
| Thavara 2009 | Outbreak of chikungunya fever in Thailand and virus detection in field population of vector mosquitoes, Aedes aegypti (L.) and Aedes albopictus Skuse (Diptera: Culicidae). | Southeast Asian J Trop Med Pub Health | Natural |
| Thenmozhi 2000 | Natural vertical transmission of dengue viruses in Aedes aegypti in southern India | Trsn Roy Soc Trop Med Hyg | Natural |
| Thenmozhi 2006 | Long-Term study of Japanese encephalitis virus infection in Anopheles subpictus in Cuddalore district, Tamil Nadu, South India | Trop Med Int Health | Natural |
| Thenmozhi 2007 | Natural vertical transmission of dengue virus in Aedes albopictus (Diptera: Culicidae) in Kerala, a southern Indian state | Jpn J Infect Dis | Natural |
| Thongrungkiat 2011 | Prospective field study of transovarial dengue virus transmission by two different forms of Aedes aegypti in an urban area of Bangkok, Thailand | J Vector Ecol | Natural |
| Turell 1982a | Transovarial and trans-stadial transmission of California encephalitis virus in Aedes dorsalis and Aedes melanimon | Am J Trop Med Hyg | Experimental |
| Turell 1982b | Stabilized infection of California encephalitis virus in Aedes dorsalis, and its implications for viral maintenance in nature | Am J Trop Med Hyg | Experimental |
| Turell 1982c | Evaluation of the efficiency of transovarial transmission of California encephalitis viral strains in Aedes dorsalis and Aedes melanimon | Am J Trop Med Hyg | Experimental |
| Turell 2001 | Vector competence of North American mosquitoes (Diptera: Culicidae) for West Nile virus | J Med Entomol | Experimental |
| Unlu 2010 | Evidence of vertical transmission of West Nile virus in field-collected mosquitoes | J Vector Ecol | Natural |
| van den Hurk 2003 | Vector competence of Australian mosquitoes (Diptera: Culicidae) for Japanese encephalitis virus | J Med Entomol | Experimental |
| Vazeille 2009 | Failure to demonstrate experimental vertical transmission of the epidemic strain of Chikungunya virus in Aedes albopictus from La Réunion Island, Indian Ocean. | Mem Inst Oswaldo Cruz | Experimental |
| Vilela 2010 | Dengue virus 3 genotype I in Aedes aegypti mosquitoes and eggs, Brazil, 2005-2006 | Emerg Infect Dis | Natural |
| Wasinpiyamongkoi 2003 | Susceptibility and transovarial transmission of dengue virus in Aedes aegypti: a preliminary study of morphological variations. | Southeast Asian J Trop Med Pub Health | Experimental |
| Watts 1973 | Transovarial transmission of La Crosse virus (California encephalitis group) in the mosquito, Aedes triseriatus | Science | Experimental |
| Watts 1974 | Overwintering of La Crosse virus in Aedes triseriatus | Am J Trop Med Hyg | Natural |
| Watts 1985 | Failure to detect natural transovarial transmission of dengue viruses by Aedes aegypti and Aedes albopictus (Diptera: Culicidae) | J Med Entomol | Natural |
| Watts 1987 | Ecological evidence against vertical transmission of eastern equine encephalitis virus by mosquitoes (Diptera: Culicidae) on the Delmarva Peninsula, USA | J Med Entomol | Natural |
| Woodring 1998 | Short report: Diapause, transovarial transmission, and filial infection rates in geographic strains of La Crosse virus-infected Aedes triseriatus | Am J Trop Med Hyg | Experimental |
| Zeidler 2008 | Dengue virus in Aedes aegypti larvae and infestation dynamics in Roraima, Brazil | Rev Saude Publica | Natural |
| Zhang 1993 | Transovarial transmission of Chikungunya virus in Aedes albopictus and Aedes aegypti mosquitoes | Chin J Virol | Experimental |
| Zhang 1996 | Transovarial transmission of Dengue viruses in Aedes albopictus and Aedes aegypti mosquitoes | Virol Sinica | Experimental |
| Zytoon 1993 | Transovarial transmission of chikungunya virus by Aedes albopictus mosquitoes ingesting microfilariae of Dirofilaria immitis under laboratory conditions | Microbiol Immunol | Experimental |
