# Supplementary material for: Determinants of Arbovirus Vertical Transmission in Mosquitoes
Source: PLoS Pathog. 2016 May 12;12(5):e1005548. doi: 10.1371/journal.ppat.1005548 (PMC4865232; doi:10.1371/journal.ppat.1005548)
Supplement: S2 Table — (DOCX) [file ppat.1005548.s002.docx]

**Database #1**

| **Factor category** | **Factors** |
| --- | --- |
|  | Publication ID |
| Virus identification | Virus species |
|  | Virus strain |
|  | Virus isolation |
| Mosquito identification | Mosquito genus |
|  | Mosquito sub-genus |
|  | Mosquito species |
|  | Mosquito strain |
|  | Mosquito-virus pair |
| Passage history of the virus | Vertebrate cell passages |
|  | Number of vertebrate cell passages |
|  | Arthropod cell passages |
|  | Number of arthropod cell passages |
|  | Alternate passage |
|  | VT history |
|  | Host type of the last passage |
|  | Complete passage history |
|  | Infection method of the parental mosquitoes |
| Was the mosquito infected by a parasite ? | Parasite (Yes or No) |
|  | Parasite species |
| Rearing conditions | Temperature |
|  | Humidity |
|  | Offspring generation tested |
|  | Ovarian cycle of tested offspring |
|  | Egg laying day after infection for tested offspring |
| Detection and identification of VT | Offspring developmental stage when VT tested |
|  | Previous cell culture before detection |
|  | Detection assay |
|  | Identification assay |
|  | VT (Yes or No) |
|  | Sample size |
|  | Minimum infection rate |

**Database #2**

| **Factor category** | **Factors** |
| --- | --- |
|  | Publication ID |
| Virus identification | Virus species |
| Mosquito identification | Mosquito genus |
|  | Mosquito sub-genus |
|  | Mosquito species |
|  | Mosquito-virus pair |
| Geographical and epidemiological context | Study site |
|  | Epidemiological context |
|  | Mosquito capture developmental stage |
| Detection and identification of VT | Mosquito developmental stage when VT tested |
|  | Previous cell culture before detection |
|  | Detection assay |
|  | Identification assay |
|  | VT (Yes or No) |
|  | Sample size |
|  | Minimum infection rate |
