# Supplementary material for: Determinants of Arbovirus Vertical Transmission in Mosquitoes
Source: PLoS Pathog. 2016 May 12;12(5):e1005548. doi: 10.1371/journal.ppat.1005548 (PMC4865232; doi:10.1371/journal.ppat.1005548)
Supplement: S3 Table — Comparison of odds ratios, confidence intervals, and p-values before and after addition of four missing publications. (DOCX) [file ppat.1005548.s003.docx]

|  | **Before (original database)** | | | | **After (addition of 4 missing publications)** | | | |
| --- | --- | --- | --- | --- | --- | --- | --- | --- |
|  | **OR** | **lower CI** | **upper CI** | ***p*-value** | **OR** | **lower CI** | **upper CI** | ***p*-value** |
| **Infection method of mothers** | | | | | | | | |
| Infectious blood meal | 1 |  |  |  | 1 |  |  |  |
| Intra-thoracic inoculation | 1.80409471908522 | 1.62906683087477 | 1.99792770544808 | < 2e-16 | 1.82399774717766 | 1.6481773798541 | 2.01857386369644 | < 2e-16 |
| Vertical transmission | 4.71292367467743 | 3.94050918348373 | 5.6367460470421 | < 2e-16 | 4.7239804147911 | 3.95132847256638 | 5.64771851144932 | < 2e-16 |
| **Detection technique** | | | | | | | | |
| Molecular | 6.8484194056505 | 0.780046071085632 | 60.1257414070633 | 0.082590 | 8.36856806246735 | 0.831286952101107 | 84.2463979966699 | 0.071367 |
| Immunological | 3.27447056459551 | 2.69706225403153 | 3.97549498991918 | < 2e-16 | 3.33442041722097 | 2.74615889568899 | 4.04869490117052 | < 2e-16 |
| Cellular | 1 |  |  |  | 1 |  |  |  |
| Animal | 0.90507964045962 | 0.276367874385298 | 2.96405346459506 | 0.869121 | 1.00012522928223 | 0.281046480308544 | 3.55902152963711 | 0.999846 |
| **Mosquito genus** | | | | | | | | |
| Aedes | 1 |  |  |  | 1 |  |  |  |
| Culex | 0.315591189875811 | 0.278192312231082 | 0.358017798293789 | < 2e-16 | 0.320006714469033 | 0.282070645002086 | 0.363044858157814 | < 2e-16 |
| **Virus genus** |  |  |  |  |  |  |  |  |
| Orthobunyavirus | 45.7088751112907 | 38.3421540505293 | 54.4909725516773 | < 2e-16 | 45.0547568357332 | 37.8163259995692 | 53.6786972259063 | < 2e-16 |
| Flavivirus | 1 |  |  |  | 1 |  |  |  |
| Alphavirus | 0.0780764676861539 | 0.013656070985515 | 0.446390093666984 | 0.004148 | 0.0508049730117657 | 0.00807175737262166 | 0.3197748846467 | 0.001500 |
| **Gonotrophic cycle** | | | | | | | | |
| First | 1 |  |  |  | 1 |  |  |  |
| Second or more | 1.65807806145314 | 1.55171185513587 | 1.7717354216073 | < 2e-16 | 1.66601830849933 | 1.5590614806298 | 1.78031273220459 | < 2e-16 |
| **Development stage of offspring** | | | | | | | | |
| Immature | 1 |  |  |  | 1 |  |  |  |
| Adult | 0.60288059411185 | 0.542920333282554 | 0.669462881522801 | < 2e-16 | 0.688722946937029 | 0.625542228961504 | 0.758285013667426 | 3.05e-14 |
